# miR-144-3p Is a Biomarker Related to Severe Corticosteroid-Dependent Asthma

**DOI:** 10.3389/fimmu.2022.858722

**Published:** 2022-04-01

**Authors:** José M. Rodrigo-Muñoz, Marta Gil-Martínez, Clara Lorente-Sorolla, Raquel García-Latorre, Marcela Valverde-Monge, Santiago Quirce, Joaquín Sastre, Victoria del Pozo

**Affiliations:** ^1^ Department of Immunology, Instituto de Investigación Sanitaria (IIS)-Fundación Jiménez Díaz, Madrid, Spain; ^2^ Centro de Investigación Biomédica en Red (CIBER) de Enfermedades Respiratorias (CIBERES), Madrid, Spain; ^3^ Department of Allergy, Instituto de Investigación Sanitaria (IIS)-Fundación Jiménez Díaz, Madrid, Spain; ^4^ Department of Allergy, Hospital La Paz-Institute for Health Research (IdiPAZ), Madrid, Spain

**Keywords:** asthma, biomarkers, epigenetics, asthma treatment, corticosteroid

## Abstract

MicroRNAs are non-coding molecules that act both as regulators of the epigenetic landscape and as biomarkers for diseases, including asthma. In the era of personalized medicine, there is a need for novel disease-associated biomarkers that can help in classifying diseases into phenotypes for treatment selection. Currently, severe eosinophilic asthma is one of the most widely studied phenotypes in clinical practice, as many patients require higher and higher doses of corticosteroids, which in some cases fail to achieve the desired outcome. Such patients may only benefit from alternative drugs such as biologics, for which novel biomarkers are necessary. The objective of the study was to study the expression of miR-144-3p in order to discover its possible use as a diagnostic biomarker for severe asthma. For this purpose, miR-144-3p was evaluated in airway biopsies and serum from asthmatics and healthy individuals. mRNA was studied in asthmatic biopsies and smooth muscle cells transfected with miR-144-3p mimic. An *in silico* regulation of miR-144-3p was performed using miRSystem, miRDB, STRING, and ShinyGO for pathway analysis. From our experimental procedures, we found that miR-144-3p is a biomarker associated with asthma severity and corticosteroid treatment. MiR-144-3p is increased in asthmatic lungs, and its presence correlates directly with blood eosinophilia and with the expression of genes involved in asthma pathophysiology in the airways. When studied in serum, this miRNA was increased in severe asthmatics and associated with higher doses of corticosteroids, thereby making it a potential biomarker for severe asthma previously treated with higher doses of corticosteroids. Thus, we can conclude that miR-144-3p is associated with severe diseases in both the airways and serum of asthmatics, and this association is related to corticosteroid treatment.

## Introduction

Asthma is a heterogeneous disease of the airways characterized by airway inflammation caused mainly by eosinophils, lymphocytes, and mast cells (1). Symptoms of asthma consist of cough, difficulty breathing, and wheezing, and specific disease manifestations are related to the inflammatory status that causes remodeling and bronchial malfunctioning (2, 3). Worsening of lung function is often related with poor responses to asthma treatment, which may indicate a requirement of a higher dose of corticosteroids, novel biological drugs, or even combined therapies (4). This clinically complicated phenotype is known as severe asthma, which carries a high economic and social burden (5), and is usually driven by eosinophilic inflammation. Patients with severe asthma require higher doses of corticosteroids, and novel biomarkers are needed to discriminate patients who present this phenotype, as doing so will facilitate clinical practice (6).

The heterogeneity of the asthmatic immune responses is a key element which has been a target of research since the first airway samples were taken until the newest technology clustering and bioinformatic analysis, depicting asthma subgroups that are related to severity, age of onset, treatment, and cellularity (7). The introduction of phenotypes, and more importantly endotypes, beneath severe asthma is one of the most important advances into how we understand asthma pathophysiology, and has been useful in defining, depending on the type of inflammation, the type 2 high severe asthma endotype (ILC2 and Th2 driven), the type 2 low endotype (neutrophilic), and the mixed endotype (T1/T2 and T2/T3 asthma) (8). Some of the differences seen between the asthmatics and the diverse phenotypes and endotypes inside this heterogeneous disease may be explained by epigenetic regulation, which acts in the development and mechanisms of asthma (9). One such mechanism involves the action of microRNAs (miRNAs), which are small non-coding RNA molecules that act as regulators of protein synthesis by gene repression, inhibiting protein translation (10), and as such are useful as biomarkers due to their high stability and relatedness to the disease (11). As part of the epigenetic posttranscriptional regulation landscape, microRNAs (including our miRNA of study: miR-144-3p) are able to modulate the pathophysiology of severe asthma, favoring the development of particular immune responses, and therefore being part of severe asthma endotypes (12).

Previous reports have described roles for miRNAs as asthma mediators and biomarkers (13, 14), and great potential has been shown for the latter (15) due to their possible use as biomarkers to facilitate asthma endotyping and phenotyping to assign treatments, as depicted in previous proof-of-concept studies (16, 17).

The objective of this study is to delve into the role of miR-144-3p, an miRNA previously related to asthma disease (18). In that first report, we observed that miR-144-3p was differentially expressed in blood eosinophils from asthmatics compared to those from controls. In this new report, we got more insight on miR-144-3p by studying its expression in the serum and lungs of asthmatics, stratifying the subjects depending on disease severity and treatment.

## Materials and Methods

### Study Subjects

Lung samples and clinical data from those patients included in this study were provided by the CIBERES Pulmonary Biobank Consortium (PT13/0010/0030), a network currently formed by twelve tertiary Spanish hospitals (www.ciberes.org) detailed in *Acknowledgements*, integrated in the Spanish National Biobanks Network, and they were processed following standard operating procedures with the appropriate approval of the Ethics and Scientific Committees. The serum samples used for this study have been described elsewhere (18) and were obtained from thirty-nine healthy controls, from ninety-six intermittent–mild–moderate asthmatics and from forty-two severe asthmatics, as diagnosed following the Spanish GEMA guidelines (19). All samples were taken at a stable state at a revision date from medical appointment. Subjects were recruited from Fundación Jiménez Díaz Hospital and La Paz University Hospital, who signed the required informed consent. The study was conducted in accordance with the Declaration of Helsinki principles and was approved by the hospital’s ethics committee.

### RNA Isolation From Biopsies, Smooth Muscle Cells, and Serum Samples

RNA was purified from lung tissue and bronchial smooth muscle cells (BSMCs) using QIAzol Lysis Reagent (Qiagen, Hilden, Germany). For gene expression analysis, 500 ng of RNA quantified by a NanoDrop ND-1000 spectrophotometer (Thermo Fisher Scientific) was reverse-transcribed with the High-Capacity cDNA Reverse Transcription Kit (Applied Biosystems, Foster City, CA, USA), followed by semiquantitative real-time PCR (qPCR) according to the manufacturer’s guidelines on a 7500 Real-Time PCR System. TaqMan™ gene expression probes were purchased for *GATA3*, *NR3C1*, *RHOA*, *PTEN*, *SOCS5*, *STAT6*, *PTGS2*, and *GAPDH* using TaqMan™ Gene Expression Master Mix (Applied Biosystems, Foster City, CA, USA).

For miRNA expression analysis in serum, RNA was first isolated from 200 µl of serum using miRNeasy Serum/Plasma Kit (Qiagen, Hilden, Germany). 200 µl of serum RNA or 20 ng of lung of BSMC RNA was retrotranscribed using the miRCURY LNA™ RT Kit (Qiagen) followed by qPCR in a LightCycler 96 thermocycler (Roche, Basel, Switzerland) using miRCURY LNA™ SYBR Green PCR Kit and miR-144-3p, miR-4251, miR-144-5p, miR-320a, miR-146b-5p, miR-146a-5p, miR-191-5p, RNU6, and UniSp6 probes (Qiagen). MiR-191-5p was used as a housekeeping miRNA for BSMCs and serum and U6 for lung biopsies. Cycle threshold (Ct) was used to calculate for relative gene expression applying the 2^-ΔΔCt^ method (20), where:

ΔΔCt=ΔCt_asthma_–ΔCt_healthy_ and ΔCt=ΔCt_gene/miRNA_–ΔCt_Housekeeping gene/miRNA_

### Cell Culture Methods

Bronchial smooth muscle cells (BSMCs) from healthy subjects (Lonza, Basel, Switzerland) were cultured in Smooth Muscle Cell Growth Medium 2 (PromoCell, Heidelberg, Germany). Medium was supplemented with 100 U/ml penicillin and 100 µg/ml streptomycin (Thermo Fisher Scientific, Waltham, MA, USA). All cultures were maintained at 37°C in an atmosphere containing 5% CO_2_.

### Transfection of miRNA Mimics and Inhibitors

Hsa-miR-144-3p miRCURY LNA miRNA Mimic (50 nM) was used to conduct overexpression experiments (MIMAT0000436: 5′UACAGUAUAGAUGAUGUACU) after purchase from Exiqon (Qiagen). Transfection of miRNA mimics was done by using TransIT-TKO^©^ Transfection Reagent, depleting the cells from serum 24 h before transfection.

### MiRNA–Gene Target and Pathway Deregulation Prediction Tools/Algorithms

MiRNA target prediction was performed using miRSystem and miRDB (21, 22). After *in silico* target genes were retrieved, pathway analysis was performed using the STRING and ShinyGO online resources (23, 24). An FDR of less than 0.05 was set.

### Statistical Analysis

Data were compared by using unpaired, two-tailed Student’s t test, or Mann–Whitney U test for non-Gaussian samples, and multiple comparisons were performed with the use of ANOVA with Bonferroni *post hoc* test or Kruskal–Wallis with Dunn test. Results are shown as mean ± SEM. *p* < 0.05 was considered as significant. Correlations were estimated by Pearson *r* or Spearman’s *ρ*. MiRNA performance as a biomarker was evaluated by a receiver operating curve (ROC).

Statistical calculations and graphs were performed with GraphPad Prism 6–8 (GraphPad Software Inc., RRID : SCR_002798, San Diego, CA).

## Results

### MiR-144-3p Is Overexpressed in Lung Biopsies From Asthmatics and Is Associated With Severity

**Figure 1:**
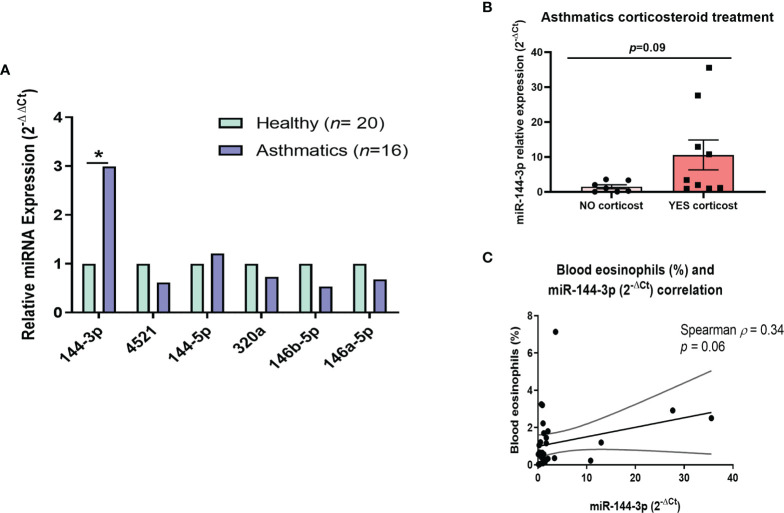
MiR-144-3p expression is increased in lung tissue from asthmatics and correlates with clinical parameters. **(A)** Relative miRNA expression (2^-ΔΔCt^) in airway biopsies from asthmatics compared to controls. **(B)** Relative miR-144-3p (2^-ΔCt^) expression in lungs from asthmatics treated with corticosteroids (YES corticost) and untreated (NO corticost). **(C)** The relative miRNA expression of miR-144-3p (2^-ΔCt^) in lung correlates inversely with blood eosinophil percentage (%). *p < 0.05.

A group of miRNAs (miR-144-3p, miR-4251, miR-144-5p, miR-320a, miR-146b-5p, and miR-146a-5p) was evaluated in lung biopsies from 16 asthmatic and 20 healthy subjects (clinical characteristics in **Table 1**). These miRNAs were selected based on our previous study (in this report, we described a differential miRNA profile in eosinophils from asthmatics compared to eosinophils from control individuals (18). Now, we studied this miRNA profile in whole lung tissue to get insight into how their expression is in the locus of inflammation, and not only in a single-cell population from blood. MiR-144-3p was differentially expressed in tissues from asthma patients (2^-ΔΔCt^ = 2.99 in asthma *versus* healthy, *p* < 0.05), while the expression of the rest of miRNAs (miR-4521, miR-320a, miR-146b-5p, miR-146a-5p, and miR-144-5p) did not change significantly (*p* > 0.05; **Figure 1A**).

**Table 1:** Demographic and clinical data of subjects from whom biopsies were obtained.

	Healthy (*n* = 20)	Asthmatics (*n* = 16)
**Age (years)** *^a^*	63.7 (46–78)	52.4 (26–79)
**Gender (female) (%)**	8 (40.0)	6 (37.5)
**Atopy (%)**	0 (0.0)	3 (18.8)
**Smoking history**		
** Current (%)**	4 (20.0)	4 (25.0)
** Ex-smoker (%)**	4 (20.0)	7 (43.7)
** Non-smoker (%)**	12 (60.0)	5 (31.3)
**Bronchiectasis (%)**	NA	2 (12.5)
**Chronic bronchitis (%)**	NA	2 (12.5)
**Immunological disease (%)**	0 (0.0)	3 (18.8)
**Cardiopathy (%)**	4 (20.0)	2 (12.5)
**Blood cell counts (%)**		
** Leukocytes (mean ± SD)**	13.7 ± 4.9	9.2 ± 3.8 *^b^*
** Neutrophils (mean ± SD)**	80.6 ± 6.1	71.4 ± 16.2 *^b^*
** Lymphocytes (mean ± SD)**	10.7 ± 3.9	18.2 ± 12.7 *^b^*
** Monocytes (mean ± SD)**	7.7 ± 2.6	8.4 ± 2.9
** Eosinophils (mean ± SD)**	0.8 ± 0.8	1.6 ± 1.9
** Basophils (mean ± SD)**	0.3 ± 0.2	0.4 ± 0.4
** Hematocrit (mean ± SD)**	35.1 ± 6.1	39.5 ± 5.6 *^b^*
** Platelets (mean ± SD)**	190.1 ± 74.2	250.4 ± 89.8 *^b^*
**FEV_1_% (mean ± SD)**	NA	69.1 ± 31.1
**FVC% (mean ± SD)**	NA	66.8 ± 28.2
**FEV_1_/FVC% (mean ± SD)**	NA	75.0 ± 15.4
**Inhaled/systemic corticosteroid treatment (%)**	NA	9 (56.3)

a Average (range).

b p < 0.05.

NA, non-available; SD, standard deviation.

When we analyzed if miR-144-3p levels were different in asthmatics depending on corticosteroid therapy (both inhaled or systemic), there could be observed a tendency for higher miR-144-3p expression in the asthmatics treated with corticosteroids compared to the untreated (2^-ΔCt^ = 10.6 ± 4.3 *vs.* 1.5 ± 0.6; *p* = 0.09; **Figure 1B**).

We subsequently analyzed the relationship of this miRNA expression (2^-ΔCt^) with clinical parameters, showing that miR-144-3p expression tends to a direct correlation with the subject’s blood eosinophil counts (Spearman *ρ* = 0.34; *p* = 0.06; **Figure 1C**). A weak inverse correlation was observed between miR-144-3p and (forced vital capacity, %) (Pearson *r* = -0.56), but the result was non-significant (*p* > 0.05). Therefore, miR-144-3p expression might be related to the severe eosinophilic asthma phenotype.

### miR-144-3p Expression in Serum Is Related to Severe Asthma and Correlates With Corticosteroid Treatment

Next, we studied if miR-144-3p expression in serum could be used as an asthma severity biomarker. To do this, we analyzed the expression data of this miRNA from a previously described cohort (18) for which the primary clinical characteristics are depicted in **Table 2**, and who were classified according to asthma severity into mild-moderate asthmatics and severe asthmatics according to GEMA guidelines (19).

**Table 2:** Demographic and clinical data of subjects from whom serum was obtained.

	Mild asthma (n = 96)	Severe asthma (n = 42)	Healthy (n = 39)
**Age (years)**	51.2 ± 16 *^a^*	60.9 ± 16 *^b^*	46.8 ± 16 *^c^*
**FEV_1_ (%)**	91.2 ± 16 *^a^*	66.3 ± 15.9	N/A
**FVC (%)**	93.8 ± 17 *^a^*	72.9 ± 20.1	N/A
**Atopy (%)**	77/96 (80) *^a^*	26/42 (62) *^b^*	3/39 (7.7) *^c^*
**Smoking history**			
** Non-smoker (%)**	62/96 (65)	32/42 (76)	16/18 (89)
** Former smoker (%)**	21/96 (22)	9/42 (22)	0/39 (0)*^b,c^*
** Smoker (%)**	13/96 (14)	1/42 (2)	2/39 (11)
**Bronchodilators**			
** Long-acting (%)**	63/96 (66) *^a^*	42/42 (100)	N/A
** B2 blockers (%)**	53/96 (55) *^a^*	34/42 (81)	N/A
**Inhaled corticoid daily dose (µg)**	427.6 ± 371.5 *^a^*	1645.7 ± 724.5	N/A
**Leukotriene receptor agonist (%)**	21/96 (22) *^a^*	30/42 (71)	N/A
**Ipratropium bromide (%)**	2/96 (2)	1/42 (2)	N/A
**Tiotropium bromide (%)**	1/96 (1) *^a^*	5/42 (12)	N/A
**Corticosteroids**			
** Oral (%)**	75/96 (78) *^a^*	42/42 (100)	N/A
** Injected (%)**	9/96 (9)	9/42 (21)	N/A
**ICU admissions (%)**	27/96 (28) *^a^*	23/42 (55)	N/A
**>5 ICU admissions (%)**	5/96 (5) *^a^*	8/42 (19)	N/A
**ACT value**	22.4 ± 3.4 *^a^*	17.8 ± 5.2	N/A
**AQL value**	5.9 ± 1.1 *^a^*	4.9 ± 1.5	N/A

NA, not available. ACT, asthma control test; AQL, Asthma Quality of Life Questionnaire.

a p < 0.05 in mild asthma versus severe asthma group.

b p < 0.05 in healthy versus severe asthma group.

c p < 0.05 in healthy versus mild asthma group.

**Figure 2:**
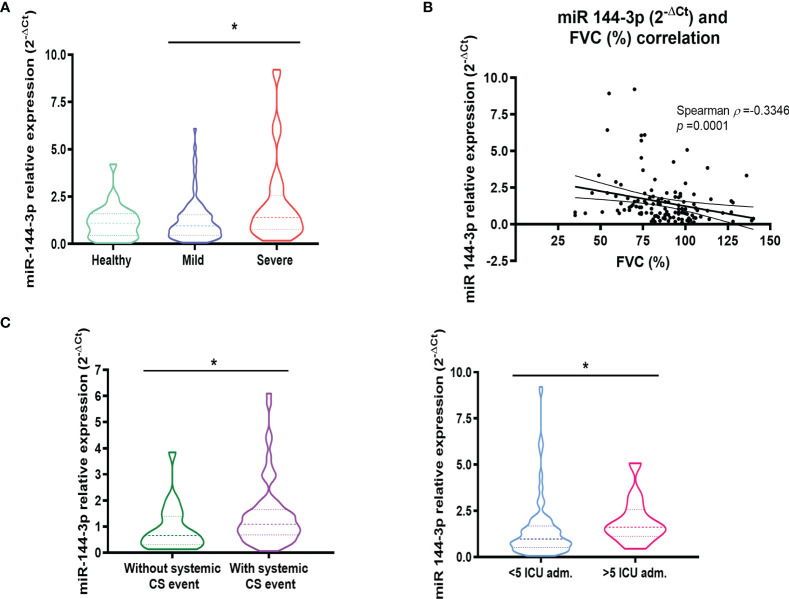
The expression of miR-144-3p in serum is related to severe asthma. **(A)** The relative miRNA expression of miR-144-3p (2^-ΔCt^) in serum is higher in severe asthmatics compared to mild to moderate asthmatics. **(B)** Relative miRNA expression of miR-144-3p (2^-ΔCt^) in serum correlates inversely with FVC (%). **(C)** miR-144-3p relative expression (2^-ΔCt^) is increased in asthmatics with a systemic corticosteroid event, and asthmatics with more than five ICU admissions. Results are shown as median and quartiles. *p < 0.05. FVC, forced vital capacity; CS, corticosteroids; ICU, intense care unit.

As a result of this analysis, we found that the expression of miR-144-3p was higher (*p* < 0.05) in severe asthmatics (2^-ΔCt^ = 2.13 ± 0.35) compared to mild to moderate asthmatic subjects (1.23 ± 0.12) as is seen in **Figure 2A**. When we compared the expression of miR-144-3p between healthy controls (2^-ΔCt^ = 1.14 ± 0.16) and mild to moderate asthmatics (1.23 ± 0.12), no differences could be observed (*p* > 0.05; **Figure 2A**). Moreover, miR-144-3p expression is higher in severe asthmatics (2^-ΔCt^ = 2.13 ± 0.35) compared to healthy (1.14 ± 0.16), although this result was statistically non-significant (*p* > 0.05; **Figure 2A**). Furthermore, miR-144-3p expression (2^-ΔCt^) inversely correlated with FVC (%). (Spearman *ρ* = -0.33; *p* < 0.05; **Figure 2B**).

The association of miR-144-3p with severe asthma was confirmed by the higher levels observed in asthmatics who needed a systemic corticosteroid treatment event (2^-ΔCt^ = 1.44 ± 0.15 *vs.* 0.90 ± 0.19; *p* < 0.05) and in asthmatics admitted to the intensive care unit (ICU) more than five times (2^-ΔCt^ = 1.95 ± 0.36 *vs.* 1.46 ± 0.15; *p* < 0.05; **Figure 2C**).

**Figure 3:**
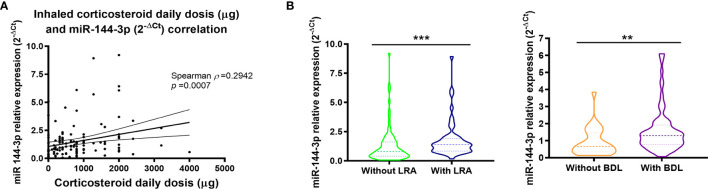
Serum miR-144-3p expression is associated with high inhaled corticosteroid dose. **(A)** Relative miRNA expression of miR-144-3p (2^-ΔCt^) in serum correlates directly with daily corticosteroid use (μg). **(B)** miR-144-3p relative expression (2^-ΔCt^) is increased in asthmatics with leukotriene receptor antagonist and bronchodilator treatment. Results are shown as median and quartiles. **p < 0.01; ***p < 0.001. LRA, leukotriene receptor antagonists; BDL, bronchodilators.

Interestingly, miR-144-3p expression directly correlates with daily inhaled corticosteroid dose (µg) for which a Spearman *ρ* of 0.29 (*p* < 0.05) was found (**Figure 3A**). Moreover, miR-144-3p is higher in asthmatics who were on leukotriene receptor antagonist treatment (LRA; 2^-ΔCt^ = 1.81 ± 0.23 *vs.* 1.32 ± 0.17; *p* < 0.05; **Figure 3B**) and in asthmatics under a bronchodilator treatment regime (BDL; 2^-ΔCt^ = 1.60 ± 0.15 *vs.* 0.92 ± 0.16; *p* < 0.05).

**Figure 4:**
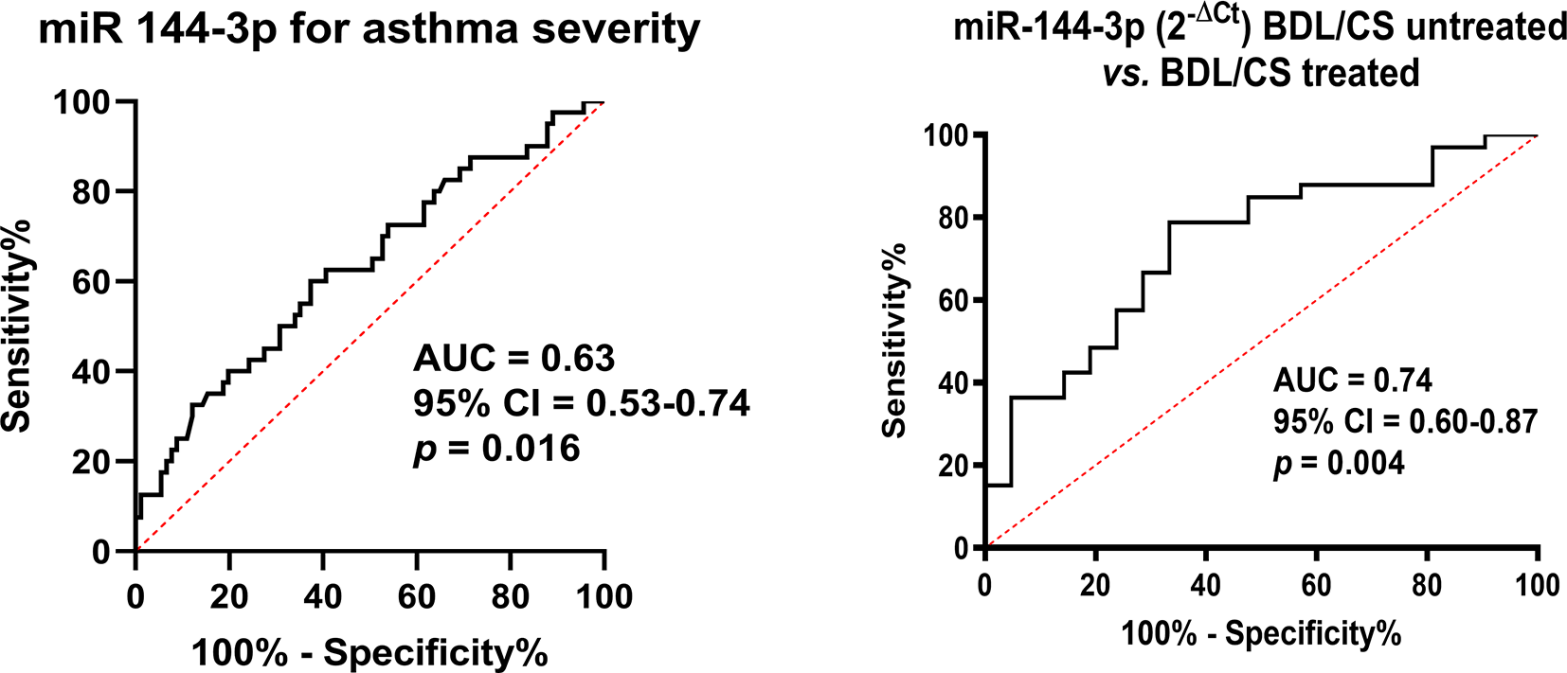
MiR-144-3p expression in serum is a biomarker for severe asthma and corticosteroid plus bronchodilator treatment. CS, corticosteroids; BDL, bronchodilators; AUC, area under the curve; CI, confidence interval.

Based on these previous results, we analyzed the usefulness of this miRNA as a possible biomarker. The expression of this miRNA in serum could be used as a modest biomarker when differentiating severe *versus* mild–moderate asthma, as we found an area under the curve (AUC) of 0.63 (*p* < 0.05; **Figure 4**). Nevertheless, miR-144-3p expression was a good serum biomarker (AUC = 0.74; *p* < 0.05; **Figure 4**) for the purpose of differentiating asthmatics under treatment with corticosteroids and bronchodilators from those with on-demand bronchodilator therapy.

### *In Silico* Analysis Reveals That miR-144-3p May Regulate Pathways Important in Asthma

To better understand the processes where miR-144-3p might be involved in asthma, we analyzed the pathways regulated by miR-144-3p *in silico*. Using the online resources miRSystem, miRDB, STRING, and ShinyGO (21–24) online resources, we observed that miR-144-3p could control biological processes of interest in asthma, including CAMP, MAPK, PI3K-Akt, TGFβ, and Ras signaling pathways, focal adhesion, and adherens junction, as seen in **Supplemental Figure 1**.

### The Expression of miR-144-3p in the Airways Directly Correlates With Genes Involved in Asthma Pathophysiology

**Figure 5:**
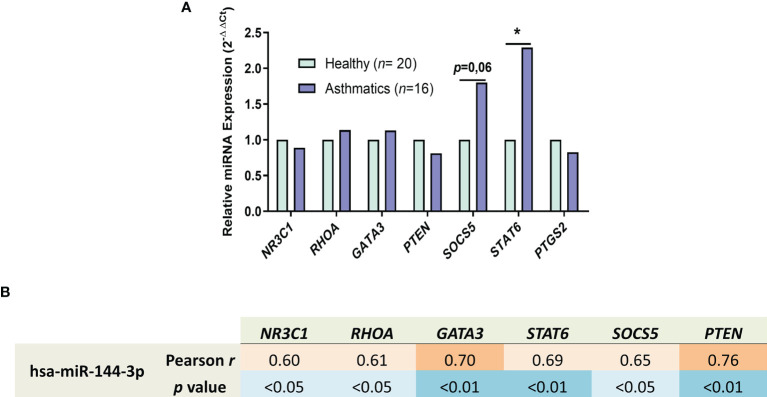
miR-144-3p expression correlates with genes involved in asthma. **(A)** Relative mRNA–gene expression (2^-ΔΔCt^) in airway biopsies from asthmatics compared to controls. **(B)** Relative miRNA expression of miR-144-3p (ΔCt) in lungs of asthmatics directly correlates with the expression of asthma involved genes (ΔCt), as shown by Pearson *r* and *p* value of the Pearson correlation test. *p < 0.05.

After we observed the biological pathways that are regulated *in silico* by miR-144-3p, we selected asthma-related target genes regulated by this miRNA from the online databases miRDB and miRSystem (21, 22) and studied its expression for better understanding how this miRNA acts in these processes. In lung biopsies from asthmatics (**Figure 5A**), *STAT6* (2^-ΔΔCt^ = 2.3; *p* < 0.05; **Figure 5A**) and *SOCS5* were highly expressed (2^-ΔΔCt^ = 1.8; *p* = 0.06). The rest of the genes, i.e., *NR3C1* (2^-ΔΔCt^ = 0.89), *RHOA* (2^-ΔΔCt^ = 1.13), *GATA3* (2^-ΔΔCt^ = 1.14), *PTEN* (2^-ΔΔCt^ = 0.81), and *PTGS2* (2^-ΔΔCt^ = 0.83), showed no differences in expression (*p* > 0.05) in samples from asthma patients *vs.* healthy subjects (**Figure 5A**).

In the asthmatic population, miR-144-3p directly correlates with *NR3C1* (Pearson *r* = 0.60; *p <*0.05), *RHOA* (Pearson *r* = 0.61; *p* < 0.05), *GATA3* (Pearson *r* = 0.70; *p* < 0.01), *STAT6* (Pearson *r* = 0.69; *p* < 0.01), *SOCS5* (Pearson *r* = 0.65; *p* < 0.05), and *PTEN* (Pearson *r* = 0.76; *p* < 0.01) as observed in **Figure 5B**, while it is not correlated with *PTGS2* expression (Pearson *r* = 0.06; *p* = 0.8, data not shown), meaning that miR-144-3p overexpression in asthmatic lungs is associated with an asthma-related genetic background.

To evaluate the possibility of a direct miRNA–target relationship for miR-144-3p with the evaluated genes, we transfected bronchial smooth muscle cells (BSMCs) with miR-144-3p mimics, causing an artificial upregulation of this miRNA. Overexpression of miR-144-3p (**Supplemental Figure 2**) only affected *GATA3* transcription factor expression (2^-ΔΔCt^ = 0.62; *p* < 0.01), having no effect (*p* > 0.05) on the remaining genes [*NR3C1* (2^-ΔΔCt^ = 0.95), *RHOA* (2^-ΔΔCt^ = 0.81), *PTEN* (2^-ΔΔCt^ = 0.96), *SOCS5* (2^-ΔΔCt^ = 0.98), *STAT6* (2^-ΔΔCt^ = 1.10), and *PTGS2* (2^-ΔΔCt^ = 1.30)].

## Discussion

This study shows the relationship of miR-144-3p in asthma pathophysiology, and its derivative use as a disease biomarker. This claim is based on the finding of increased expression of miR-144-3p in lung biopsies and serum from asthmatics compared to controls.

Furthermore, the expression of miR-144-3p in bronchial samples from asthmatic subjects correlated directly with the expression of genes involved in asthma pathogenesis such as *GATA3*, *STAT6*, *SOCS5*, *RHOA*, *NR3C1*, and *PTEN. GATA3*, which was previously reported to be higher in airways from asthmatics (25); *STAT6*, a key molecule required for eotaxin production and pulmonary eosinophilia (26); *SOCS5*, which has been previously defined as an inductor of airway eosinophilia (27); *RHOA*, a kinase with the ability to control variable cell functions deregulated in asthma including proliferation, recruitment, and contraction (28); *NR3C1*, which is the coding gene for the corticosteroid receptor whose single-nucleotide polymorphisms (SNPs) are associated with reduced corticosteroid effect over TGF-β (29); and *PTEN*, a phosphatase that has been related to the successful treatment of airway inflammation and hyperresponsiveness (30).

The correlation observed between the expression of these genes and miR-144-3p may be indirect, resulting in the overexpression of these genes in asthmatic lung tissue. Having in mind that *SOCS5* is a suppressor of cytokine signaling that is able to regulate negatively the activation of *STAT6* induced by IL-4, these two genes may be involved in a negative feedback loop to control cytokine synthesis (31). Besides, miR-144-3p overexpression in bronchial smooth muscle cells only affected *GATA3* mRNA, and previous reports have shown that the reduction of *GATA3* by SB010 DNAzyme attenuated allergic asthma responses by type 2 helper T-cell (Th2) reduction (32), so in this sense, miR-144-3p may behave as an inhibitor of *GATA3* in a similar manner and may even control signaling pathways involved in asthma pathophysiology as observed *in silico*. Nonetheless, this observation should be confirmed in further *in vitro* and *in vivo* severe asthma models, to get a complete insight into the role of miR-144-3p in severe asthma.

Several studies have attempted to use miRNAs for asthma severity determination in biofluids. MiR-28-3p, miR-16-2-3p, and miR-210-3p have recently been associated with severe asthma and were suggested as biomarkers, although the study was only preliminary (33). Other reports have elucidated profiles of miRNAs in sputum related to neutrophilic severe asthma, of which miR-223-3p was found to be a key node (17). In addition, our research group has previously described miRNA profiles that could be helpful in classifying asthmatics according to the severity of the disease, in discrimination of the eosinophilic phenotype, or for the prediction of the response to biological drugs (16, 18, 34, 35). This new report on miR-144-3p adds new knowledge of miRNAs in severe eosinophilic asthma, reporting another miRNA that can help in unravelling the complex epigenetic landscape of this disease.

The association of miR-144-3p with severe asthma was confirmed in serum, as miR-144-3p levels were higher in the severe asthmatic group compared to both the mild asthma group and healthy controls, showing that on the systemic level the expression of this miRNA is increased among patients with the most severe disease. These data are interesting as, in our previous study, we did not find any difference between healthy and asthmatics serum miR-144-3p expression, but indeed, when stratifying the asthmatics into severity phenotype, the difference can be seen (18). This highlights the importance of stratifying the asthmatic patients depending on different phenotypes or endotypes, as with this, some previously hidden characteristics can be found.

The relationship between miR-144-3p and severe asthma is further supported by the inverse correlation observed between miR-144-3p and FVC, which implies that the higher miR-144-3p is in serum, the worse the lung function, possibly due to higher airway obstruction (36). The relationship between miR-144-3p and asthma severity is also reflected in the direct correlation observed with daily inhaled corticosteroid doses. Additionally, a higher expression of miR-144-3p was found in asthmatic subjects who required a systemic corticosteroid treatment event, in subjects with more than five visits to the ICU, and in subjects treated with leukotriene receptor antagonist; all of these events are related to severe asthma, with high corticosteroid treatment and poor treatment response. Many of these subjects may benefit from a more personalized treatment for their severe asthma such as biological drugs, which can reduce asthma symptoms and corticosteroid dosage, thereby indicating that miR-144-3p expression analysis may help in identifying these patient candidates (37).

In accordance with our results, in another study, miR-144-3p was found increased in mouse lungs upon dexamethasone treatment (38), and indeed, we found that miR-144-3p expression in the lungs has a tendency for higher levels in asthmatics under corticosteroid treatment. Inside the group of asthmatics under corticosteroid treatment, there could be observed what seems to be two small clusters inside it, with four patients having the highest miR-144-3p expression. Although this group is very small, a possibility exists that there are asthmatics with very high miR-144-3p expression in their lungs that may comprise a different endotype inside the corticosteroid-treated asthmatics. Nevertheless, further experimentation and studies are needed in this matter, as from our results we cannot associate this small endotype to any other clinical characteristic of asthma. This raises the question of whether the increase of miR-144-3p derives from the severity of asthma or from the high corticosteroid dose used to treat asthmatic subjects (39). This question will likely be of interest for future studies, although in the meantime it is noteworthy that the expression of miR-144-3p in the serum of asthmatic patients can be used as a biomarker for asthma subphenotyping, as seen in the ROC curve analysis, where the expression of this miRNA in serum had an AUC of 0.63 for differentiating severe asthmatics form mild to moderate asthmatics (being a mildly poor biomarker), and an AUC of 0.74 when distinguishing asthmatics who are in treatment with corticosteroids and bronchodilators from those with on-demand bronchodilator therapy (being a good biomarker for asthma treatment). The importance of biomarkers for asthma diagnosis has been previously shown, as lung function tests are not always good, and therefore combinatorial biomarkers seem to be the most promising diagnostic tool in asthma (40).

Moreover, the possible increase of miR-144-3p in the lungs and serum due to corticosteroid treatment, as observed in mouse lung tissue (38), and based on the direct correlation observed between corticosteroid dose and miR-144-3p expression, might be an indicator of corticosteroid use and treatment adherence and thus might be helpful in determining which patients are truly taking the treatment. This information might help in isolating those asthmatics that are corticosteroid responders from those who might need higher doses or who may benefit from an alternative therapy (41).

By contrast, it is worth noticing that miR-144-3p expression in eosinophils from asthmatic patients is lower than healthy eosinophils (18). This can be explained by different reasons, as the study was done in peripheral eosinophils; furthermore, eosinophils normally comprise around >3% of the asthmatic airways cellularity (42), and lung tissue is a complex network with many cell types interacting, and thus, the expression of a miRNA can be divergent between the whole tissue and a cell type that is part of it. This has been previously shown in other tissues like the brain, where miRNAs have different expressions and effects depending on the cell type (43).

There are some limitations in our study: one is the absence of a clinical classification of lung tissue into severity, although a trend toward a higher miR-144-3p expression could be observed in asthmatic lungs of subjects treated with inhaled or systemic corticosteroids, which may indicate an association to severe asthma; the second is that the small number of lung samples can be blurring both the correlation results, as the expression of miR-144-3p in biopsies seems to correlate inversely with FCV, and the trend for increased miR-144-3p expression in corticosteroid-treated asthmatics, having non-statistically significant results, which therefore cannot be thoroughly confirmed. Nevertheless, tendency for a direct correlation was observed between miR-144-3p expression in lung and the counts of blood eosinophils and thus together with the FCV correlation may indicate that the asthmatic subjects with higher miR-144-3p expression in their lungs present the severe eosinophilic asthma phenotype.

In conclusion, these results highlight the importance of miRNAs in asthmatic disease, from their involvement in pathophysiology by means of modulation of key pathways to their use in diagnosis as biofluid markers. In this study, we found that miR-144-3p is upregulated in lung biopsies from asthmatic subjects compared to healthy individuals and that miR-144-3p expression is correlated with worst lung function and with type 2 inflammatory profiles in blood. Furthermore, miR-144-3p is increased in severe asthmatics treated with higher doses of corticosteroids, thus opening new possibilities for asthma diagnosis and treatment.

## Data Availability Statement

The raw data supporting the conclusions of this article will be made available by the authors, without undue reservation.

## Ethics Statement

The studies involving human participants were reviewed and approved by Comite de Etica de la Investigacion de la Fundacion Jimenez Diaz. The patients/participants provided their written informed consent to participate in this study.

## Author Contributions

VP conceived the manuscript and designed the study. JR-M performed the experiments and data analysis and had primary responsibility for writing the manuscript. MG-M, CL-S, and RG-L performed the experiments and obtained data. MV-M, SQ, and JS recruited patients and obtained samples and clinical data. All authors contributed to the article and approved the submitted version.

## Funding

This work was supported by the Fondo de Investigación Sanitaria PI18/00044, FI16/00036, and FI19/00067; Ciber de Enfermedades Respiratorias (CIBERES); RTC-2017-6501-1 (Ministerio de Ciencia, Innovación y Universidades); and FEDER funds (Fondo Europeo de Desarrollo Regional).

## Conflict of Interest

S.Q. reports personal fees from AstraZeneca, Novartis, Sanofi, Boehringer Ingelheim, Teva, ALK, Mundipharma, GSK, Chiesi, and personal fees from Leti, outside the submitted work. J.S. reports having served as a consultant to Thermofisher, MEDA, Novartis, Sanofi, Leti, Faes Farma, Mundipharma, and GSK; having been paid lecture fees by Novartis, GSK, Stallergenes, Leti, and Faes Pharma; as well as having received grant support for research from Thermofisher, Sanofi, and ALK. V.d.P reports having served as a consultant to Astra Zeneca and GSK and having been paid lecture fees by both.

The remaining authors declare that the research was conducted in the absence of any commercial or financial relationships that could be construed as a potential conflict of interest.

## Publisher’s Note

All claims expressed in this article are solely those of the authors and do not necessarily represent those of their affiliated organizations, or those of the publisher, the editors and the reviewers. Any product that may be evaluated in this article, or claim that may be made by its manufacturer, is not guaranteed or endorsed by the publisher.

## Supplementary Material

Supplementary Figure 1 In silico gene enrichment analysis and representation of the pathways regulated by miR-144-3p (FDR<0.05), with a possible role in asthma pathophysiology. Lines imply connection and networking, thicker edges represent more overlapping genes, more intense blue nodes are more significantly enriched gene sets and bigger nodes are larger gene sets. FDR, False discovery rate.

Supplementary Figure 2 MiR-144-3p upregulation with artificial miRNA MIMICs in bronchial smooth muscle cells reduces GATA3 expression (2^-ΔΔCt^). **p<0.01. BSMC, bronchial smooth muscle cells.
