# Supplementary figures and images for: miR-144-3p Is a Biomarker Related to Severe Corticosteroid-Dependent Asthma
Source: Front Immunol. 2022 Apr 1;13:858722. doi: 10.3389/fimmu.2022.858722 (PMC9010740; doi:10.3389/fimmu.2022.858722)

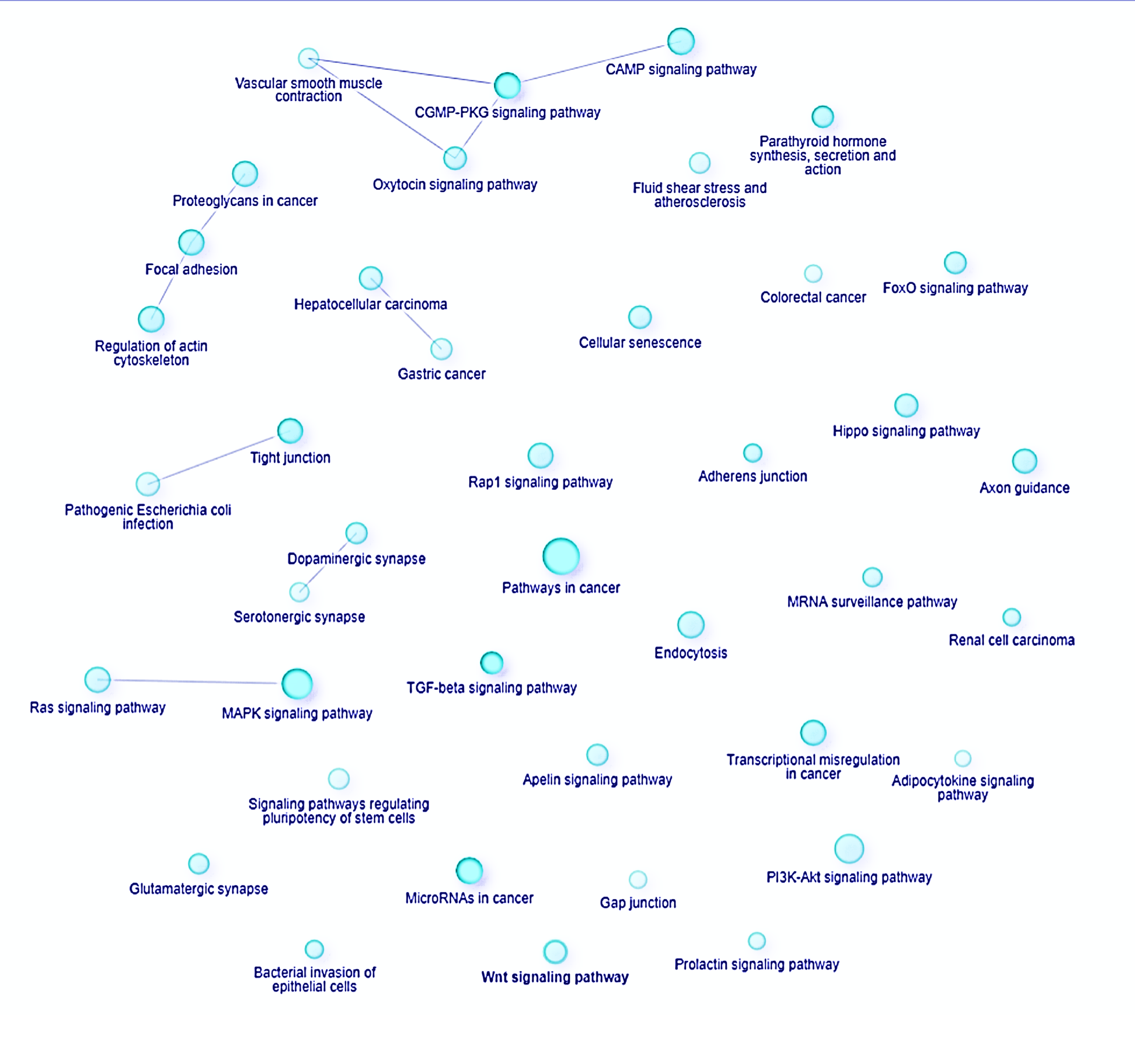

Supplement: Supplementary Figure 1 — In silico gene enrichment analysis and representation of the pathways regulated by miR-144-3p (FDR<0.05), with a possible role in asthma pathophysiology. Lines imply connection and networking, thicker edges represent more overlapping genes, more intense blue nodes are more significantly enriched gene sets and bigger nodes are larger gene sets. FDR, False discovery rate. [file Image_1.tif]

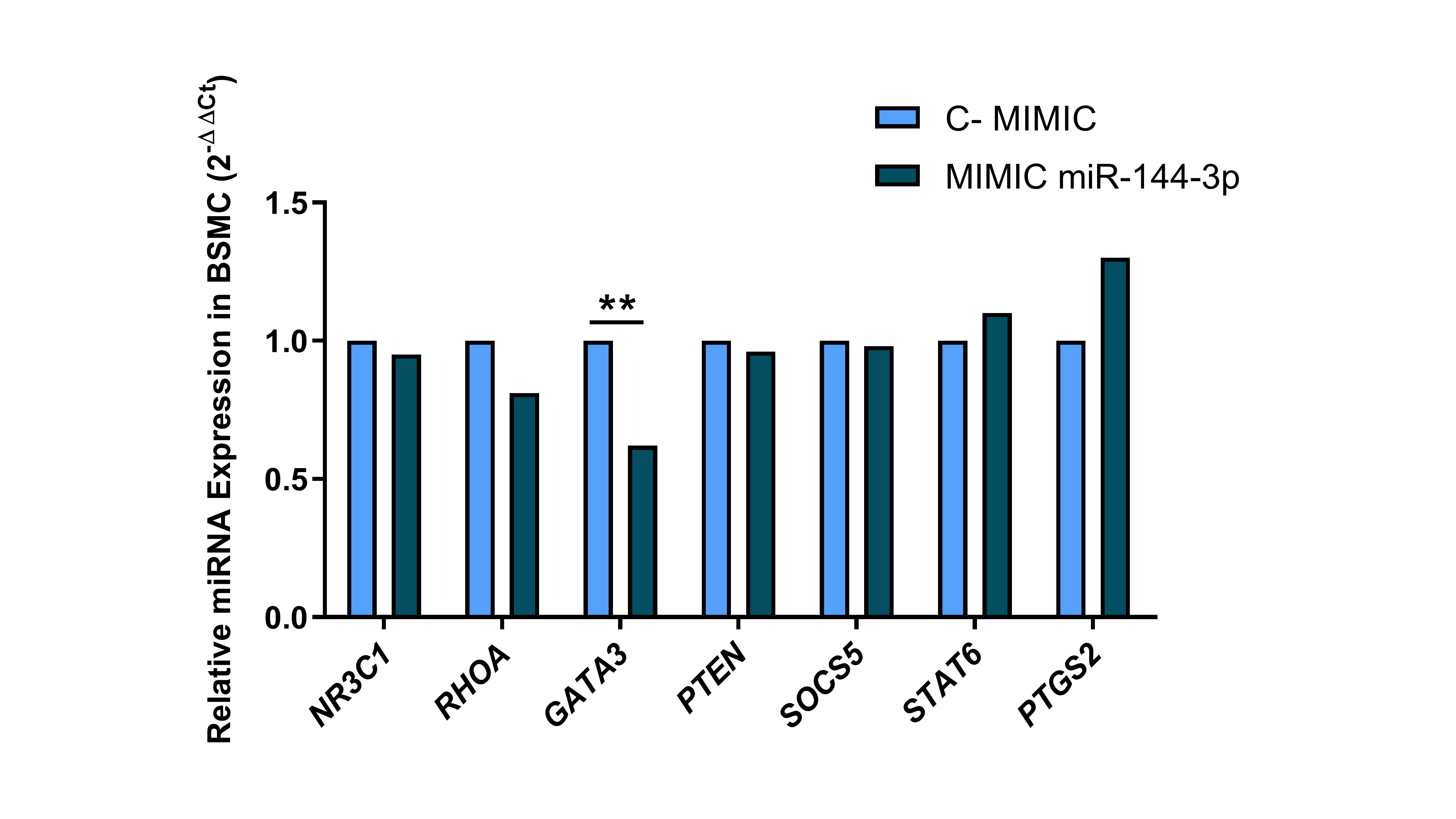

Supplement: Supplementary Figure 2 — MiR-144-3p upregulation with artificial miRNA MIMICs in bronchial smooth muscle cells reduces GATA3 expression (2-ΔΔCt). **p<0.01. BSMC, bronchial smooth muscle cells. [file Image_2.tif]
